# Reduced responsiveness of blood leukocytes to lipopolysaccharide does not predict nosocomial infections in critically ill patients

**DOI:** 10.1186/cc14118

**Published:** 2015-03-16

**Authors:** LA Van Vught, MA Wiewel, AJ Hoogendijk, BP Scicluna, H Belkasim, J Horn, MJ Schultz, T Van der Poll

**Affiliations:** 1 Academic Medical Center, Amsterdam, the Netherlands

## Introduction

Critically ill patients show signs of immune suppression, which is considered to increase vulnerability to nosocomial infections. Whole blood stimulation is a frequently used functional test for immune suppression. We here aimed to assess the association between whole blood leukocyte responsiveness to lipopolysaccharide (LPS) and the subsequent occurrence of nosocomial infections in critically ill patients admitted to the ICU.

## Methods

All consecutive critically ill patients admitted to the ICU between April 2012 and June 2013 with two or more systemic inflammatory response syndrome criteria and an expected length of ICU stay of more than 24 hours were enrolled. Age-matched and gender-matched healthy individuals were included as controls. Blood was drawn the first morning after ICU admission and stimulated *ex vivo *with 100 ng/ml ultrapure LPS for 3 hours. Tumor necrosis factor (TNF)-α, interleukin (IL)-1β and IL-6 were measured in supernatants.

## Results

**Figure 1:**
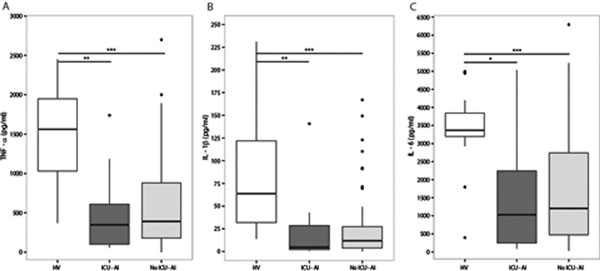
**Similarly reduced responsiveness of whole blood leukocytes to lipopolysaccharide**.

Seventy-three critically ill patients were included, 10 of whom developed an ICU-acquired infection. Compared with healthy subjects, whole blood leukocytes of patients were less responsive to *ex vivo *stimulation with LPS, as reflected by strongly reduced TNFα, IL-1β and IL-6 levels in culture supernatants. However, results were not different between patients who did and those who did not develop an ICU-acquired infection (Figure 1).

## Conclusion

The extent of reduced LPS responsiveness of blood leukocytes in critically ill patients on the first day after ICU admission does not relate to the subsequent development of ICU-acquired infections.

